# Sex‐specific immune cell signatures define clinical stages and severity of Alzheimer's disease

**DOI:** 10.1002/alz70856_103630

**Published:** 2025-12-24

**Authors:** Aaron Burberry, Mark J Lowe, Wanyong Shin, Blake McCourt, Holden Maecker, Lynn M. Bekris, Quintasha Beamon, Sara Ramaiah, Elizabeth Woidke, Maria Khrestian, William S Bush, Penelope Benchek, Stephen M. Rao, James B Leverenz, Jagan A. Pillai

**Affiliations:** ^1^ Case Western Reserve Univeristy, Cleveland, OH, USA; ^2^ Cleveland Clinic, Cleveland, OH, USA; ^3^ Stanford University, Stanford, CA, USA; ^4^ Cleveland Clinic Genomic Medicine Institute, Cleveland, OH, USA; ^5^ Department of Population and Quantitative Health Sciences, Case Western Reserve University School of Medicine, Cleveland, OH, USA; ^6^ Cleveland Clinic Lou Ruvo Center for Brain Health, Las Vegas, NV, USA

## Abstract

**Background:**

Immune‐related changes impact clinical outcomes in people with Alzheimer's disease (AD). However, these changes have yet to translate into robust blood biomarkers of AD, in part due to high inter‐individual variation, small effect sizes, and the indirect relationship between peripheral blood and central nervous system changes.

**Method:**

In this prospective cohort study of 55 subjects, 19 healthy controls (11 female and 8 male), 19 mild cognitive impairment (MCI) stage of AD (13 female and 6 male), 8 AD dementia (4 female and 4 male) and 9 frontotemporal lobar degeneration (FTD) (7 female and 2 male) individuals, we used mass cytometry to identify a sex‐specific immune cell signature in AD peripheral blood and cerebrospinal fluid (CSF). Dynamic contrast enhanced magnetic resonance (DCE‐MRI) imaging was used to assess regional blood‐brain barrier (BBB) permeability in subjects. Five patients contributed blood and CSF samples after one‐year longitudinal follow‐up.

**Result:**

Peripheral blood immune cell responsiveness is more robust than the CSF immune cell signature and peaks earlier in the peripheral blood in the MCI stage among females and later in males at the AD dementia stage. To demonstrate clinical utility of this immune cell signature to identify the MCI stage of AD, we show its effect size exceeded that of Amyloid β42/40, phospho‐tau181, plasma phospho‐tau217, and cytokine signatures both in the plasma and CSF and was significantly elevated relative to FTD. The immune cell activation signature also correlated with MRI measures of hippocampal BBB permeability and cognitive outcomes.

**Conclusion:**

The immune cell changes identified here were distinct among cellular phenotypes in peripheral blood and CSF in their responsiveness to AD pathological changes and show promise for novel biomarkers and future immune related therapeutics in AD.

